# Local perceptions of causes of death in rural South Africa: a comparison of perceived and verbal autopsy causes of death

**DOI:** 10.3402/gha.v8.28302

**Published:** 2015-07-17

**Authors:** Laith Hussain-Alkhateeb, Edward Fottrell, Max Petzold, Kathleen Kahn, Peter Byass

**Affiliations:** 1 Health Metrics, Sahlgrenska Academy, University of Gothenburg, Gothenburg, Sweden; 2 Institute for Global Health, University College London, London, UK; 3 WHO Collaborating Centre for Verbal Autopsy, Umeå Centre for Global Health Research, Umeå University, Umeå, Sweden; 4 Wits University Rural Public Health, School of Public Health, Faculty of Health Sciences, University of the Witwatersrand, Johannesburg, South Africa; 5 INDEPTH Network, Accra, Ghana; 6 Medical Research Council, Johannesburg, South Africa; 7 Wits University Rural Public Health and Health Transitions Research Unit (Agincourt), School of Public Health, Faculty of Health Sciences, University of the Witwatersrand, Johannesburg, South Africa

**Keywords:** community perception, causes of death, verbal autopsy

## Abstract

**Background:**

Understanding how lay people perceive the causes of mortality and their associated risk factors is important for public health. In resource-limited settings, where verbal autopsy (VA) is used as the most expedient method of determining cause of death, it is important to understand how pre-existing concepts of cause of death among VA-informants may influence their VA-responses and the consequential impact on cause of death assessment. This study describes the agreement between VA-derived causes of death and informant-perceived causes and associated influential factors, which also reflects lay health literacy in this setting.

**Method:**

Using 20 years of VA data (*n*=11,228) from the Agincourt Health and Demographic Surveillance System (HDSS) site in rural South Africa, we explored the agreement between the causes of death perceived by the VA-informants and those assigned by the automated Inter-VA tool. Kappa statistics and concordance correlation coefficients were applied to measure agreement at individual and population levels, respectively. Multivariable regression models were used to explore factors associated with recognised lay perceptions of causes of mortality.

**Results:**

Agreement between informant-perceived and VA-derived causes of death at the individual level was limited, but varied substantially by cause of death. However, agreement at the population level, comparing cause-specific mortality fractions was higher, with the notable exception of bewitchment as a cause. More recent deaths, those in adults aged 15–49 years, deaths outside the home, and those associated with external causes showed higher concordance with InterVA.

**Conclusion:**

Overall, informant perception of causes of death was limited, but depended on informant characteristics and causes of death, and to some extent involved non-biomedical constructs. Understanding discordance between perceived and recognised causes of death is important for public health planning; low community understanding of causes of death may be detrimental to public health. These findings also illustrate the importance of using rigorous and standardised VA methods rather than relying on informants’ reported causes of death.

Lay opinions about health and disease reflect conceptual models applied by individuals or communities to derive reasons for ill-health and causes of death. Examining divergence between lay perceptions of health and disease and established medical convention can illuminate modern health challenges and inequalities (1). An holistic approach to understanding health and disease also supports strategies set by national and international health decision makers for enhancing self-management of health and empowers people to seek out and handle information responsibly (2, 3).

In resource-poor settings, assessing how people make appropriate health decisions at home or at work, in the community, in the health system, and the political arena (4) – an overall concept of ‘health literacy’ – requires good understanding of how lay people perceive health, illness, and causes of death. In South Africa, as in other nations, emerging health problems, epidemics, and chronic illnesses mean that understanding people's perceptions and their health-related behaviour is particularly essential for effective medical practice and public health interventions (2, 5, 6). In this African context, different cultural and ethnic groups may disagree about symptomatology of particular diseases (1). These disagreements can advance into multifarious conceptual models about health and related causes of mortality across different groups. Consequently, community perceptions of disease processes may diverge from those of health professionals (1, 7). Swami et al. suggested that some communities tend to describe their poor health inconsistently with medical evidence, but such views persist since they form part of the conceptual knowledge of that community (1). Employing traditional methods to assess community understanding of related causes of mortality in Africa has resulted in a broad spectrum of outcomes (8–12). Nonetheless, no studies have yet sought standardised tools such as the verbal autopsy (VA) to explore people's perception of causes of death in African settings, which is the intention of this research.

VA is a widely used approach for ascertaining causes of death in settings that lack complete physician certification (13, 14). Trained fieldworkers use standardised interviews with informants (usually caretakers or close relatives of the deceased) to gather information on the signs, symptoms, and circumstances of death, and this information is then used to identify a probable medical cause of death. The automated InterVA-4 tool, which has been validated in several settings, has shown substantial benefits over physician reviews, particularly its consistency over time and place (15, 16).

The VA method relies to a large extent on lay informants to elicit a specific cause of death. Informant-given responses to VA questions can be influenced by the informant's own perception of causes of death which could ultimately impact on the InterVA assessment of cause of death (14). Evidence of relying on lay reports as a basis for national estimates of cause-specific mortality is not yet proven, with both lay reports and physicians failing to distinguish between common causes of deaths such as TB and HIV/AIDS in some instances (17).

Using 20 years of VA data from the Agincourt Health and Socio-Demographic Surveillance System (HDSS) site in South Africa, this study aims to describe the agreement between objective VA-derived causes of death with the informant-perceived causes gathered during the VA interview. The study also explores informant-related background and socio-economic factors contributing to the derived level of agreement whereby public health implications of misperception of causes of mortality in the rural South African context are also addressed.

## Methods

### Study population and context

In this empirical study, data were generated from longitudinal observations covering the period from March 1992 to December 2011 in the Agincourt HDSS, in Mpumalanga Province, South Africa. The Agincourt HDSS, described in detail elsewhere (18), has been operating as a monitoring system for around 90,000 inhabitants living in approximately 16,000 households across 26 villages, since 1992. The HDSS procedures for capturing vital events [births, deaths, in-and out-migrations, and other individual characteristics such as marital status, nationality, education, and socio-economic status (SES)] also include VA interviews to establish likely causes of death.

### Verbal autopsy

During this 20-year period, 12,206 deaths in the Agincourt HDSS were routinely followed-up by the trained fieldworkers to conduct VA with next of kin or caregiver, and VAs were successfully completed in 11,228 cases (92.0%). Standardised interviews in the local language (Shangaan) were used to elicit signs, symptoms, and circumstances of the terminal illness. During the VA interviews, interviewers also sought respondents’ opinions about the causes of death (5). We refer to this here as the ‘respondent-reported cause of death’ (RRCoD).

Until fairly recently, VA material was usually interpreted into causes of death by presenting individual interview data to local physicians (physician-certified verbal autopsy, PCVA) (19). However, to address time, cost, and reliability issues associated with PCVA (9), computerised approaches that are faster, cheaper, and more consistent have been developed and are now in widespread use (20). The most widely used is InterVA, a Bayesian-based model using a probability matrix to generically estimate the most likely cause(s) of death from VA data (21). Developed from a combination of existing data and input from an expert panel, InterVA-4 is freely available resource (www.interva.net) which is fully compliant with the WHO 2012 VA standard (22). Equivalence between InterVA-4 cause of death assignments and the PCVA approach has also been demonstrated in a large-scale international study (23). Within the Agincourt HDSS, the VA archives from 1992 have been retrospectively processed using InterVA-4 (24). We refer to this source here as the ‘verbal autopsy cause of death’ (VACoD).

### Data management

HDSS data on the deceased's background and characteristics were taken directly from the Agincourt database. The recorded deaths were categorised into four age groups (0–14, 15–49, 50–64, and 65+years), two origins (in-migrants from Mozambique and their direct descendants, or South African), and three levels of education measured by the numbers of completed years of study (none, 1–7, and 8–15 years). Relationship between the VA respondent and the deceased was classified in six groups (parent, grandparent, spouse, child, sibling, and other non-relatives). Other HDSS information pertaining to the main dwelling, sanitation, power, and livestock (all with given ordinal scores) was handled to create a SES factor representing household wealth in quintiles. Individual level data from the VA interviews comprising details on any previous illness that had occurred, duration of the final illness (acute:<2 weeks and chronic:≥2 weeks), cumulative number of deaths in the household (one death, two deaths, and three or more deaths at one household), time (1992–1996, 1997–2001, 2002–2006, and 2007–2011), and place of death (home, hospital/health facility, or other) were recorded. Any therapeutic approach prior to death (western or traditional, or both) and the RRCoD was also retrieved from the VA interview data (5).

### Exploring agreement at individual level

Each individual VA was processed using InterVA-4 model which assigns up to three likely causes of death per WHO VA cause categories (22). RRCoDs were given as a single cause in most cases. For comparing RRCoD and VACoD, a total of 17 causes of death groups were generated including the indeterminate group, as listed in Table 1. Both the RRCoD and VACoD data were re-categorised into these 17 groups; individual agreement was achieved for any case where there was an RRCoD and VACoD category in common. Cohen's kappa statistic (κ) was used to compare the proportions of agreement between two readings which are made by two different approaches, that is, InterVA and RRCoD, taking into account the proportion of agreement that would be expected due to chance.

**Table 1:** Proportions of deaths as assigned to VACoD, for 6,721 deaths in the Agincourt HDSS, South Africa, with RRCoD and 4,507 without RRCoD

	RRCoD given		RRCoD not given	
		
Cause of death category	*n*	%	*n*	%
Accidental causes	394	5.9	48	1.1
Homicide or suicide	356	5.3	102	2.3
Nervous system disease	30	0.4	6	0.1
Diarrhoeal disease	175	2.6	69	1.5
Maternal causes	23	0.3	15	0.3
Indeterminate	368	5.5	282	6.3
HIV/TB	2,579	38.4	2,078	46.1
Stroke	224	3.3	63	1.4
Cardiovascular disease	256	3.8	156	3.5
Neoplasm	416	6.2	457	10.1
Endocrine or malnutrition	186	2.8	133	3.0
Neonatal or congenital	166	2.5	124	2.8
Chronic liver disease	29	0.4	36	0.8
Chronic respiratory disease	273	4.1	182	4.0
Acute infectious disease	221	3.3	124	2.8
Acute respiratory disease	870	12.9	521	11.6
Other	159	2.4	111	2.5

VACoD=verbal autopsy causes of death; RRCoD=respondent-reported cause of death.

The binomial agreement (agreement or not) between VACoD and RRCoD was analysed against the informant's background, characteristics, and socio-economic factors for each cause of death category using multivariable logistic regression with a *p*-value cut-off point of 0.05. The multivariable regression model building followed a ‘forward’ selection approach carried on the selected variables of interest. The likelihood-ratio test was performed to compare the log likelihood of the two models every time a new variable was added and best model-fit was therefore retained. Missing values in both ‘illness duration’ and ‘therapeutic approach’ factors were coded ‘unknown’ since they considerably refer to accidental, homicide, or suicides.

### Estimating agreement at population level

Population-level cause-specific mortality fractions (CSMF) were derived from the InterVA-4 output to estimate the proportion that each cause of death category contributed to the total number of deaths (5). Differences between proportions and 95% confidence intervals of those differences were calculated for each cause of death group between VACoD and RRCoD. A concordance correlation coefficient (CCC) for the CSMFs from VACoD and RRCoD was also computed using Lin's CCC with 95% CI (25). Based on Pearson's correlation, the CCC quantifies the agreement between the two measures (23).

### Ethical clearance

The study data were obtained from the Agincourt HDSS where on-going ethical clearance has been granted by the University of Witwatersrand's Committee for Research on Human Subjects (No. M960720 & M110138). The principle of informed consent was fully respected with the right for refusal or withdrawal from interviews at both individual and household levels. No specific additional ethical clearance was required for this study.

## Results

Of 11,228 deaths for which a VA was successfully completed, InterVA-4 assigned a single cause of death to 9,434 individuals (84.0%), two likely causes to 1,108 individuals (9.9%), and three likely causes to 36 cases (0.3%). There were a further 650 cases (5.8%) specifically designated ‘indeterminate’ by InterVA-4, generally reflecting a lack of specific VA data or contradictory evidence. Of the 11,228 cases with VA findings, 6,721 (59.9%) recorded specific RRCoDs (including ‘do not know’ reports, but excluding reiteration of symptoms that were not actually causes of death) during VA interviews. Table 1 shows the VACoD distribution for both VA interviews where respondents did or did not report a cause of death.

For the VA interviews where RRCoD was reported, 6,645 (98.8%) gave a single cause (of which 2,292 were ‘do not know’), 73 (1.1%) reported two causes, and three (0.1%) reported 3 or 4 causes. Overall, there was agreement on at least one cause between RRCoD and VACoD in 2,080 cases (30.9%). Table 2 shows VACoD and RRCoD by cause category, together with the agreement between RRCoD and VACoD, and the associated kappa statistic. Kappa (κ)>0.4 is considered moderate-to-good agreement (26). Bewitchment as RRCoD was reported for 865 cases, which had no possibility of being designated as a VACoD cause. Over 40% of the bewitchment cases were associated with HIV/TB as a VACoD.

**Table 2:** Proportions of deaths with individual agreement between VACoD and RRCoD, for 6,721 deaths in the Agincourt HDSS, South Africa

Cause of death category	VACoD *n*	RRCoD *n*	Agreement% (95% CI)	Kappa statistic (κ) (95% CI)
Accidental causes	407	557	79.1 (75.2–83.1)	0.64 (0.61–0.68)
Homicide or suicide	367	358	69.8 (65.0–74.4)	0.69 (0.65–0.73)
Nervous system disease	32	127	53.1 (35.6–70.7)	0.21 (0.12–0.29)
Diarrhoeal disease	186	464	45.2 (38.0–52.3)	0.23 (0.18–0.27)
Maternal causes	23	18	34.8 (14.9–54.7)	0.39 (0.20–0.58)
Indeterminate	374	2,292	34.5 (29.6–39.4)	0.001 (−0.013–−0.016)^a^
HIV/TB	2,857	903	29.6 (27.9–31.4)	0.30 (0.28–0.32)
Stroke	252	211	27.4 (21.9–32.9)	0.27 (0.22–0.33)
Cardiovascular disease	301	264	22.1 (17.4–26.8)	0.20 (0.15–0.25)
Neoplasm	476	254	19.2 (15.7–22.9)	0.21 (0.16–0.25)
Endocrine or malnutrition	215	197	13.3 (8.7–17.9)	0.11 (0.06–0.16)
Neonatal or congenital	171	39	11.2 (6.5–16.0)	0.18 (0.11–0.25)
Chronic liver disease	36	46	8.6 (−0.8–18.0)^a^	0.07 (−0.01–−0.15)^a^
Chronic respiratory disease	282	67	5.8 (3.1–8.6)	0.08 (0.04–0.12)
Acute infectious disease	247	83	4.5 (1.9–7.1)	0.05 (0.01–0.09)
Acute respiratory disease	953	27	1.1 (0.4–1.8)	0.01 (0.002–0.02)
Other	189	28	1.1 (−0.4–2.5)^a^	0.01 (−0.01–0.04)^a^
Bewitchment	–	865	–	–
Total	4,514	4,510	–	–

Individual deaths can contribute to more than one category, where so described by InterVA-4 or the respondent's report.

a Not significantly different from zero agreement. VACoD=verbal autopsy causes of death; RRCoD=respondent-reported cause of death.

Results from a multivariable logistic regression model of background factors in relation to agreement between RRCoD and VACoD are presented in Table 3. Increased accurate perception of causes of mortality was associated with deaths of people aged 15–49 years, deaths in more recent time periods, deaths with longer illness durations (or those with unknown illness duration, which on closer inspection were largely accidents, homicides, and suicides), and deaths which did not occur at home.

**Table 3:** Background characteristics for 6,721 deaths in the Agincourt HDSS, South Africa, showing multivariable OR for agreement between RRCoD and VACoD, including ‘unknowns’

Background characteristics		Number of deaths (%)	OR (95% CI)
Age group	0–14 (child)	1,322 (19.7)	Ref
	15–49 (reproductive age)	2,560 (38.1)	1.46 (1.15–1.86)^a^
	50–64 (adult)	1,429 (21.3)	1.06 (0.81–1.38)
	>65 (elder)	1,405 (20.9)	0.98 (0.74–1.29)
Sex	Male	3,573 (53.2)	Ref
	Female	3,148 (46.8)	1.04 (0.93–1.17)
Origin	Mozambican origin	2,061 (30.7)	Ref
	South African origin	4,660 (69.3)	1.09 (0.96–1.24)
Respondent	Child	867 (12.9)	Ref
	Parent	2,418 (36.0)	0.89 (0.72–1.10)
	Spouse/sibling	2,132 (31.7)	1.12 (0.92–1.36)
	Other	1,304 (19.4)	1.16 (0.95–1.42)
Years of education	None	2,707 (32.8)	Ref
	1–7	1,465 (21.8)	1.14 (0.96–1.34)
	8–15	1,591 (23.7)	1.11 (0.92–1.34)
	Other/too young	1,458 (21.7)	0.86 (0.68–1.09)
Period of death	1992–1996	766 (11.4)	Ref
	1997–2001	1,161 (17.3)	1.03 (0.83–1.29)
	2002–2006	1,802 (26.8)	1.26 (1.02–1.55)^a^
	2007–2011	2,992 (44.5)	1.21 (0.99–1.48)
Cumulative household deaths	1 Person	2,744 (40.8)	Ref
	2 People	2,137 (31.8)	1.01 (0.89–1.15)
	3 People or more	1,840 (27.4)	1.07 (0.93–1.22)
Illness duration	<2 weeks (acute)	1,530 (22.8)	Ref
	>2 weeks (chronic)	4,026 (59.9)	1.16 (1.01–1.34)^a^
	Unknown	1,165 (17.3)	3.59 (2.96–4.34)^a^
Previous illness	No	4,522 (67.3)	Ref
	Yes	2,199 (32.7)	1.12 (0.98–1.28)
Therapeutic approach	None	775 (11.5)	Ref
	Western only	2,576 (38.3)	1.14 (0.93–1.40)
	Traditional only	164 (2.4)	0.67 (0.43–1.06)
	Western and traditional	2,457 (36.6)	0.98 (0.79–1.21)
	Unknown	749 (11.1)	1.23 (0.97–1.55)
Place of death	Home	2,795 (41.6)	Ref
	Hospital	3,071 (45.7)	1.18 (1.04–1.34)^a^
	Other locations	855 (12.7)	1.56 (1.30–1.88)^a^

a Odds ratio significantly different from unity. OR=odds ratio; VACoD=verbal autopsy causes of death; RRCoD=respondent-reported cause of death.

**Fig. 1:**
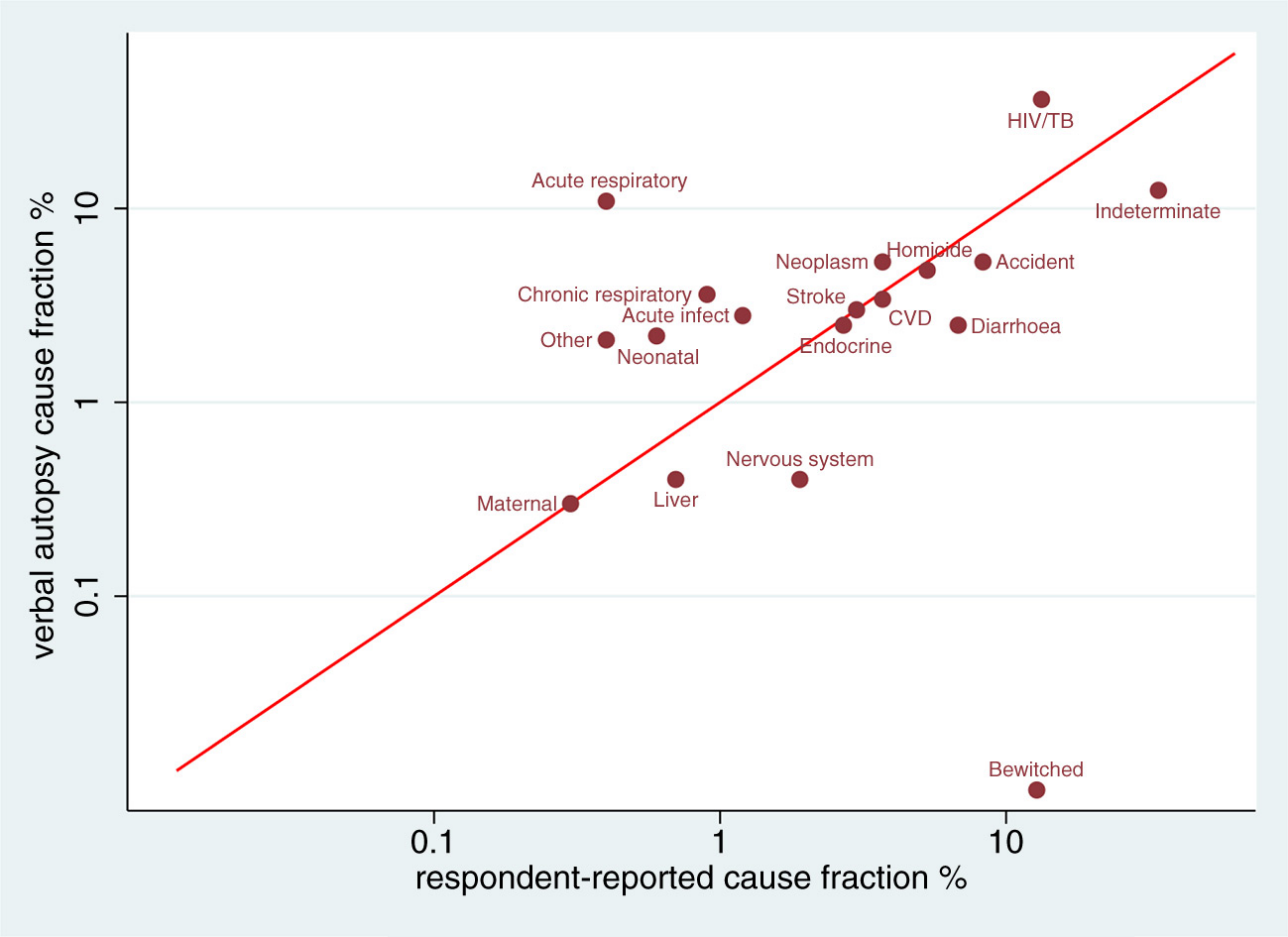
Concordance (log-log scale) between CSMF determined by VA and respondent reports, in relation to the line of equivalence, for 6,721 deaths in the Agincourt HDSS, South Africa. CSMF=cause-specific mortality fractions; VA= verbal autopsy.

To assess agreement at the population level, CSMFs derived from the VA using the InterVA-4 models were compared with those obtained from RRCoD as presented in Table 4, together with the CSMF difference and 95% confidence interval. Concordance between the VACoD and RRCoD fractions is shown in Fig. 1, with a CCC of 0.43 (95% CI: 0.04–0.83; *p*=0.03).

**Table 4:** CSMF from VACoD and RRCoD for 6,721 deaths in Agincourt HDSS, South Africa

	Cause-specific mortality fraction		
	
Cause of death category	VACoD%	RRCoD%	Difference% (95% CI)
HIV/TB	36.5	13.3	−23.2 (−24.6–−21.8)^a^
Indeterminate	12.4	34.1	21.7 (20.3−23.1)^a^
Acute respiratory disease	10.9	0.4	−10.5 (−11.3−9.7)^a^
Neoplasm	5.3	3.7	−1.5 (−2.2−0.8)^a^
Accidental causes	5.3	8.3	3.0 (2.2−3.8)^a^
Homicide or suicide	4.8	5.3	0.5 (−0.2−1.2)
Chronic respiratory disease	3.6	0.9	−2.7 (−3.2−2.2)^a^
Cardiovascular disease	3.4	3.7	0.3 (−0.3−0.9)
Stroke	3.0	3.0	0.1 (−0.5−0.6)
Acute infectious disease	2.8	1.2	−1.6 (−2.1−1.1)^a^
Endocrine or malnutrition	2.5	2.7	0.2 (−0.3−0.7)
Diarrhoeal disease	2.5	6.8	4.3 (3.6−5.0)^a^
Neonatal or congenital	2.2	0.6	−1.6 (−2.0−1.2)^a^
Others	2.1	0.4	−1.7 (−2.1−1.3)^a^
Chronic liver disease	0.4	0.7	0.3 (0.1−0.6)^a^
Nervous system disease	0.4	1.9	1.5 (1.1−1.9)^a^
Maternal	0.3	0.3	0.0 (−0.2−0.2)
Bewitched	0.0	12.8	12.8 (12.0−13.6)^a^

a Difference significantly different from zero. CSMF=cause-specific mortality fractions; VACoD=verbal autopsy causes of death; RRCoD=respondent-reported cause of death.

## Discussion

In this African setting, where most deaths pass undocumented or unattended by professionals, reliable information on cause of death is scarce (27). The relatively robust nature of the InterVA-4 tool, and particularly its consistency over time and place, made it the choice for the reference group here (15, 16, 21, 28, 29). During the early part of the period covered by this dataset, a massive epidemic of HIV/AIDS occurred in Agincourt, which subsided latterly as HIV treatment options took effect (16). This may well have influenced local perceptions of causes of death over time (30). Interestingly, Table 1 shows that the RRCoDs were less likely to be given for deaths due to HIV/TB, neoplasm, or acute respiratory diseases, perhaps reflecting a degree of stigmatisation or the entrenched cultural beliefs with respect to some causes.

Exploring pre-existing concepts about causes of deaths among VA respondents is crucial for documenting mortality and understanding patterns of death for public health planning (14). Moreover, it reflects on how local beliefs may influence care-seeking, public health prevention, and impact of health care service interventions in this setting. Not least, a VA respondent's perceptions of cause of death may sway responses to specific questions in a VA interview, and thereby affect conclusions on cause of death. The limited perception of causes of mortality revealed here, with only a fairly small proportion of respondents providing a medically plausible cause of death, is not surprising and may also be similar in other national or regional African settings (31, 32). African countries have some of the lowest literacy rates in the world, particularly among females, though South Africa has some of the highest literacy rates in the continent, at around 80% (33). How literacy affects the ability to read, understand, and act on healthcare information – holistic concept of health literacy (34) – is a more complex issue. The amount of medical information available to VA respondents is also likely to vary with the circumstances of death. Causes for deaths which occurred at home were reported less reliably than those occurring in hospital, as shown in Table 3.

The comparability of the derived causes of death categories at the individual level between both approaches reflected a broad range of agreements. Not surprisingly, causes such as accidents, homicides, and suicides, associated with minimal needs for understanding underlying biomedical processes, were more reliably reported by VA respondents. In a large proportion of cases, VA respondents simply stated that they did not know any cause of death, in much greater numbers than were assigned by VA to be indeterminate. In many of the cases excluded from the overall dataset, for which no eligible cause of death (including ‘do not know’) was given by the respondent, a major symptom of the final illness was reported instead. This appears to reflect a lack of understanding about the concept of cause of death, as distinct from observed symptoms, reinforcing the importance of standardised VA methods for determining cause of death. Perceptions about connections between symptoms and disease processes that may lead to death can cause inconsistency in recognising causes of death (35). The substantial proportion of deaths attributed to witchcraft in this population also emphasises that non-biomedical models of thinking may be applied in many cases (5).

Perhaps surprisingly, there were relatively lower levels of agreement on respiratory and infectious causes. Studies elsewhere have suggested that pneumonia, particularly in childhood, can achieve good lay recognition, and it is unclear why that might not be the case here (31, 32). The pluralistic behaviour in seeking healthcare in the Agincourt area reflects the need for a systematic measure in assessing individuals’ acts throughout the course of illness, which may ultimately explore peoples’ misunderstandings of disease processes (36, 37).

The HIV/TB cause category was the leading cause of death during the study period, and the epidemic dynamics, together with substantial stigmatisation, certainly contributed to understandings and expressions of cause of death, both for adults and children (38). This was partly reflected in the tendency to attribute HIV/AIDS mortality to witchcraft. Other research in the Agincourt reported death attributed to witchcraft was 70% higher in children than adults (5). HIV/AIDS, and by association tuberculosis and pneumonia, may be considered socially sensitive conditions in which people may favour secrecy. In a random survey of 2,500 residents, in the town of Carletonville, South Africa, participants were offered a free and anonymous HIV test, but not one accepted (39). Another study showed that 92% of the 726 HIV-positive patients had not told anyone about their status (40). Anti-retroviral therapies were not as widely available through the public health services, albeit stigmatisation and the concept that people with AIDS are ‘dead before dying’ contributed to more than 80% of South Africans needing anti-retroviral drugs to still be without treatment by 2006 (41).

South Africa remains in the midst of a profound health transition shaped by changing burdens of communicable, non-communicable, perinatal, maternal, and external causes of death. During the past 20 years of political transition – which coincides with the time span of this dataset – South Africa has seen the rise of non-communicable diseases driven by increases in related risk factors (42). The behavioural and social determinants of non-communicable diseases and their associated risk factors are important (43), and may be a key area for health education campaigns.

VA techniques are generally applied to ascertain cause of death patterns in communities where there is insufficient health infrastructure for all deaths to be routinely and consistently assigned a cause by physicians. Although VA has its limitations, it has been shown on a large scale that standardised VA interviews with automatic processing of causes of death achieve substantially equivalent findings to VAs assessed by physicians (44). While this study into VA respondents’ own perceptions of causes of death is interesting, it is also clear that this source provided a much less complete and consistent picture of cause-specific mortality. For national decision makers and epidemiologists, this study emphasises the importance of applying rigorous and consistent VA methods rather than relying on individual opinions (21, 22). Translating cause-specific mortality data into effective policy making remains an on-going challenge (45).
